# Analysis of the Patient-Physician Relationship, Race, and Pain Control and Physical Function Among Adults With Chronic Low Back Pain

**DOI:** 10.1001/jamanetworkopen.2022.16270

**Published:** 2022-06-09

**Authors:** John C. Licciardone, Sweta Ganta, Leah Goehring, Kendall Wallace, Ryan Pu

**Affiliations:** 1 University of North Texas Health Science Center, Fort Worth

## Abstract

**Importance:**

Racial and ethnic disparities in pain outcomes are widely reported in the United States. However, the impact of the patient-physician relationship on such outcomes remains unclear.

**Objective:**

To determine whether the patient-physician relationship mediates the association of race with pain outcomes.

**Design, Setting, and Participants:**

This cross-sectional study uses data from the Pain Registry for Epidemiological, Clinical, and Interventional Studies and Innovation, collected from April 2016 to December 2021. All registry enrollees who identified as Black or White with chronic low back pain who had a regular physician who provided pain care were included. Data were analyzed during December 2021.

**Exposures:**

Participant-reported aspects of their patient-physician relationship, including physician communication, physician empathy, and satisfaction with physician encounters.

**Main Outcomes and Measures:**

The primary outcomes included low back pain intensity, measured with a numerical rating scale and physical function, measured with the Roland-Morris Disability Questionnaire. Mediator variables were derived from the Communication Behavior Questionnaire, Consultation and Relational Empathy measure, and Patient Satisfaction Questionnaire.

**Results:**

Among 1177 participants, the mean (SD) age was 53.5 (13.1) years, and there were 876 (74.4%) women. A total of 217 participants (18.4%) were Black, and 960 participants (81.6%) were White. The only difference between Black and White participants in the patient-physician relationship involved effective and open physician communication, which favored Black participants (mean communication score, 72.1 [95% CI, 68.8-75.4] vs 67.9 [95% CI, 66.2-69.6]; *P* = .03). Black participants, compared with White participants reported worse outcomes for pain intensity (mean pain score, 7.1 [95% CI, 6.8-7.3] vs 5.8 [95% CI, 5.7-6.0]; *P* < .001) and back-related disability (mean disability score, 15.8 [95% CI, 15.1-16.6] vs 14.1 [95% CI, 13.8-14.5]; *P* < .001). In mediation analyses that controlled for potential confounders using disease risk scores, virtually none of the associations of race with each outcome was mediated by the individual or combined factors of physician communication, physician empathy, and patient satisfaction. Similarly, no mediation was observed in sensitivity analyses that included only participants with both chronic low back pain and the same treating physician for more than 5 years.

**Conclusions and Relevance:**

These findings suggest that factors other than the patient-physician relationship were important to pain disparities experienced by Black participants. Additional research on systemic factors, such as access to high-quality medical care, may be helpful in identifying more promising approaches to mitigating racial pain disparities.

## Key Points

**Question:**

Does the patient-physician relationship mediate the association between race and chronic pain outcomes?

**Findings:**

This cross-sectional study among 1177 participants in a national pain research registry found significantly worse outcomes among Black vs White participants for pain intensity and disability. In mediation analyses, essentially none of the associations of race with either outcome was attributable to physician communication, physician empathy, or patient satisfaction.

**Meaning:**

These findings suggest that factors other than the patient-physician relationship, such as lack of access to high-quality medical care, are likely related to pain disparities experienced by Black patients.

## Teaser

This cross-sectional study assesses whether the patient-physician relationship mediates the association of race with pain control and physical function among adults with chronic low back pain.

## Introduction

Questions regarding race and the patient-physician relationship have been raised in the medical literature for almost 50 years.^1^ In the intervening period, programs have been introduced with the aims of improving access to and quality of medical care for Black individuals in the US. However, many questions regarding race and the patient-physician relationship remain largely unanswered today.^2^ Moreover, the degree to which the patient-physician relationship affects racial health disparities remains unclear. Pain is one area wherein racial health disparities have been documented.^3^ The National Pain Strategy recommends improving communication between patients and physicians to eliminate such disparities and promote equity in pain assessment and treatment.^4^ The Federal Pain Research Strategy further identifies pain disparities as an important area of inquiry, with investigative priorities involving the effect of race on pain incidence, treatment, and outcomes.^5^ The Work Group on the Prevention of Acute and Chronic Pain recommends implementation research to understand how patient and physician beliefs and behaviors affect the long-term success of interventions aimed at reducing the intensity and impact of chronic pain.^6^

The patient-physician relationship, including physician communication and physician empathy, is particularly important in conditions involving chronic pain.^7^ It is generally thought that a collaborative approach involving both the patient and physician in clinical decision making improves patient adherence to pain treatment and its outcomes. This has been demonstrated in inpatient and outpatient orthopedic rehabilitation centers providing multimodal treatment programs for patients with chronic low back pain.^8^ The interpersonal aspects of the patient-physician relationship, including patient-centered communication, may facilitate and enhance patient engagement in chronic pain self-management.^9^ Physician empathy also has potential implications for pain disparities, based on emerging research involving neurophysiologic activity in the anterior insula and anterior cingulate cortex and biobehavioral studies.^7^ In a study by Kaseweter et al,^10^ a group of predominantly White nurses were asked to make pain treatment recommendations and to rate their empathic reactions after viewing video clips of Black and White patients matched according to facial pain expression. The nurses recommended more aggressive pain treatment for White patients, which was correlated with higher ratings of empathy for those patients. Similar findings were observed in a study involving White medical students and residents who rated 15 true or false statements about biological differences between Black and White patients and then read mock cases involving a kidney stone or ankle fracture among both Black and White patients.^11^ About one-half of participants reported that at least 1 false statement about racial differences was true. Moreover, participants who endorsed such false beliefs also rated the pain intensity of the Black patients lower than that of the White patients and made less accurate pain treatment recommendations.

Such studies suggest that racial biases exist among physicians. These may affect the physician’s interpretation of the patient pain experience, interaction with patients, treatment plans, and outcomes. The aims of this study were to measure racial differences in the patient-physician relationship and in pain and related physical function outcomes among adults with chronic low back pain, and to determine whether the patient-physician relationship helps explain the associations of race with outcomes.

## Methods

This cross-sectional study was approved by the North Texas Regional Institutional Review Board, and a current registry overview is available elsewhere.^12^ All study participants provided either written or electronic informed consent and reported data on a series of validated pain research and related instruments at their initial registry encounter. This study is reported following the Strengthening the Reporting of Observational Studies in Epidemiology (STROBE) reporting guideline.

### Research Setting

This study was conducted within the Pain Registry for Epidemiological, Clinical, and Interventional Studies and Innovation. The registry was established in 2016 at the University of North Texas Health Science Center and has since expanded to cover the 48 contiguous states and District of Columbia through a digital research platform. The registry recruits participants using direct-to-the-patient advertising, rather than relying on referrals from physicians.

### Study Design

This cross-sectional study was conducted from April 2016 to December 2021. It included registry participants aged 21 to 79 years with chronic low back pain and a physician who regularly treated that pain. Chronic low back pain was assessed using criteria recommended by the National Institutes of Health Task Force on Research Standards for Chronic Low Back Pain.^13^ These required having low back pain for at least the past 3 to 6 months and with a frequency of at least half of the days over the past 6 months. Persons were excluded from the study if they reported being pregnant, residing in an institutional facility, or being in a racial category other than Black or White. Participants could also report on Hispanic ethnicity.

### Participant Characteristics

Comprehensive sociodemographic, psychological, and clinical data were self-reported by study participants at registry enrollment.^12^ These included elements of the minimum data set recommended by the National Institutes of Health Task Force on Research Standards for Chronic Low Back Pain,^13^ and data derived from such validated research instruments as the Patient-Reported Outcomes Measurement Information System with 29 items,^14^ Pain Catastrophizing Scale,^15^ and Pain-Self Efficacy Questionnaire.^16^ A history of medical conditions inventory was used to collect data on 9 common spinal, medical, or mental health diagnoses. The current use of opioids for low back pain was also measured.

### Measures of the Patient-Physician Relationship

The patient-physician relationship was assessed using 3 validated research instruments. The Communication Behavior Questionnaire included 23 items that provided scores on 4 scales of patient perception of physician communication.^8,17^ These include patient participation and patient orientation, effective and open communication, emotionally supportive communication, and communication about personal circumstances. Scores range from 0 to 100 on each scale, with higher scores representing better perceived physician communication. The Consultation and Relational Empathy measure included 10 items that provided a score for patient perception of physician empathy.^18^ Scores range from 10 to 50, with higher scores indicating greater perceived physician empathy. The Patient Satisfaction Questionnaire included 18 items that provided scores for 7 aspects of patient satisfaction with medical care.^19^ The 5 scales assessed in this study were those inherently related to the patient-physician relationship: general satisfaction with medical care, technical quality of the physician, interpersonal manner of the physician, physician communication, and time spent with the physician. Scores range from 1 to 5 on each scale, with higher scores representing greater patient satisfaction. Scores on the Consultation and Relational Empathy measure and on each scale of the Patient Satisfaction Questionnaire were linearly transformed to range from 0 to 100 to facilitate direct comparisons across all instruments and scales that assessed the patient-physician relationship.

### Outcome Measures

Chronic low back pain outcomes were measured using a numerical rating scale for pain intensity and the Roland-Morris Disability Questionnaire (RMDQ).^20^ Pain intensity was rated on a scale from 0 (no pain) to 10 (worst pain), representing the average pain level over the 7 days prior to registry enrollment. The RMDQ included 24 items that asked participants about the adverse impact of low back pain on physical function at the time of registry enrollment. Scores range from 0 to 24, with higher scores representing greater back-related disability. The numerical rating scale for pain intensity and RMDQ are among the most commonly used outcome measures for low back pain, and both are recommended by the National Institutes of Health Task Force on Research Standards for Chronic Low Back Pain.^13^

### Statistical Analysis

The characteristics of Black and White participants were compared using contingency table methods for categorical variables and student *t* test for continuous variables. The student *t* test was also used to compare racial groups on each aspect of the patient-physician relationship and both outcome measures. Cohen *d* was used to further identify clinically important findings (defined as *d* ≥ 0.2).^21^

Mediation analysis was used to determine whether the patient-physician relationship helped explain the associations between race and each outcome measure. These analyses were performed with PROCESS software^22^ version 4.0, using multiple mediation models. Race was entered as the independent variable (*X*), physician communication (*M*_1_), physician empathy (*M*_2_), and patient satisfaction (*M*_3_) were each entered as mediators (in combination representing the mediator vector), and low back pain intensity (*Y*_1_) and back-related disability (*Y*_2_) were the outcome variables. The regression coefficient (*c*) for the total effect of race with each outcome variable was modeled as the regression coefficient (*c*’) for the direct effect of race plus the sum of the 3 regression coefficients (*a_i_b_i_*) for the indirect effect attributable to the mediator vector. Because the measures of physician communication and patient satisfaction involved multiple scales, a mean score was computed for each mediator based on its component scales.

The mediation models also included a disease risk score to adjust for potential confounding variables. The disease risk score for low back pain intensity was computed using a multiple logistic regression model for the risk of severe low back pain intensity, which was defined as a numerical rating scale score of 7 or greater. This cut point was based on a commonly used rating system for mild, moderate, and severe low back pain and selected 482 participants (41.0%) having severe low back pain intensity. Correspondingly, the disease risk score for physical function was computed for the risk of severe back-related disability, defined as a RMDQ score of 17 or greater. This cut point selected 489 participants (41.5%) having severe back-related disability. The multiple logistic regression models used to compute these disease risk scores involved 20 independent variables, including age, gender, ethnicity, educational level, cigarette smoking status, duration of low back pain, pain catastrophizing, pain self-efficacy, history of comorbid conditions (herniated disk, sciatica, osteoarthritis, osteoporosis, hypertension, heart disease, diabetes, asthma, and depression), health-related quality of life, prior surgery for low back pain, and current use of opioids for low back pain. Each mediation model used 10 000 resamples to compute 95% bootstrap CIs. Two sensitivity analyses were performed. The first excluded the disease risk score as a covariate in the mediation models. The second used the disease risk score but included only participants with both chronic low back pain and the same treating physician for more than 5 years to assess long-term patient-physician relationships.

The study sample size was sufficient to detect virtually all clinically important racial differences (ie, *d* ≥ 0.21) pertaining to the patient-physician relationship and clinical outcomes with 80% statistical power. The registry’s digital research platform precludes submission of case report forms with missing data. All analyses were performed with the SPSS Statistics software package version 28 (IBM). Hypotheses were assessed at the .05 level of significance using 2-sided tests. Data were analyzed during December 2021.

## Results

**Figure 1.**
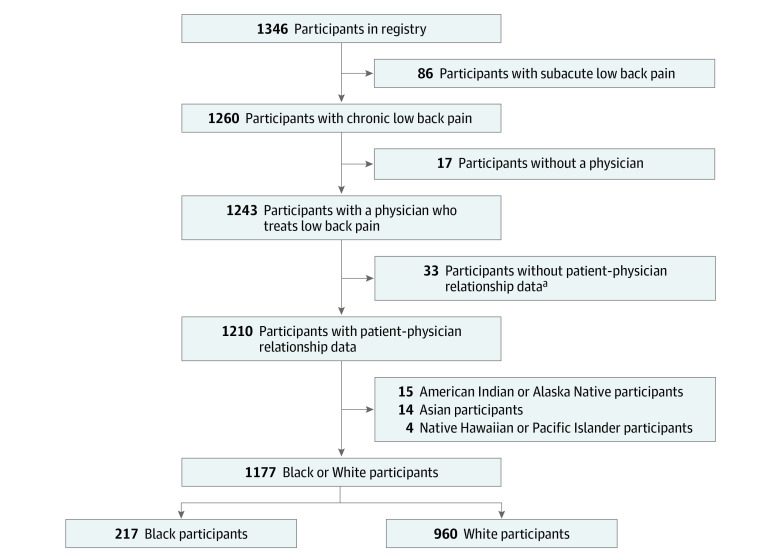
Flowchart of Participants Through the Study ^a^Registry data on the patient-physician relationship were not collected prior to September 2016.

Among a total of 1177 participants, the mean (SD) age was 53.5 (13.1) years, and there were 876 (74.4%) women. Stratified by race, there were 217 Black participants (18.4%) and 960 White participants (81.6%) (Figure 1). Black and White participants differed on many sociodemographic, psychological, and clinical characteristics (Table), including age (mean [SD], 50.9 [12.4] years vs 54.1 [13.2] years), pain catastrophizing (mean [SD] score, 24.3 [14.7] vs 18.4 [12.7]), having at least a college education (odds ratio [OR], 0.50 [95% CI, 0.36-0.70]), current cigarette smoking (OR, 2.43 [95% CI, 1.73-3.42]), having low back pain for more than 5 years (OR, 0.59 [95% CI, 0.43-0.79]), having had surgical treatment for low back pain (OR, 0.35 [95% CI, 0.22-0.58]), having received workers’ compensation or disability payments for low back pain (OR, 1.72 [95% CI, 1.24-2.38]), and having a history of sciatica (OR, 0.35 [95% CI, 0.26-0.49]) or osteoarthritis (OR, 0.44 [95% CI, 0.32-0.61]). There was no significant difference in use of opioids for low back pain (OR, 1.33; 95% CI, 0.98-1.80).

**Table.** Participant Characteristics According to Racial Group

Characteristic	Participants, No. (%)		*P* value
	Black (n = 217)	White (n = 960)	
Age, mean (SD), y	50.9 (12.4)	54.1 (13.2)	.001
Gender			
Women	150 (69.1)	726 (75.6)	.047
Men	67 (30.9)	234 (24.4)	
Ethnicity			
Hispanic	9 (4.1)	89 (9.3)	.01
Non-Hispanic	208 (95.9)	871 (90.7)	
Educational level			
<College degree	158 (72.8)	551 (57.4)	<.001
≥College degree	59 (27.2)	409 (42.6)	
Cigarette smoking status			
Never or former smoker	153 (70.5)	819 (85.3)	<.001
Current smoker	64 (29.5)	141 (14.7)	
BMI, mean (SD)	34.1 (8.5)	32.5 (8.4)	.01
Duration of low back pain, y			
≤5	88 (40.6)	274 (28.5)	<.001
>5	129 (59.4)	686 (71.5)	
History of low back surgery			
No	198 (91.2)	755 (78.6)	<.001
Yes	19 (8.8)	205 (21.4)	
Presence of chronic widespread pain			
No	148 (68.2)	717 (74.7)	.051
Yes	69 (31.8)	243 (25.3)	
Work loss ≥1 mo owing to low back pain			
No	109 (50.2)	575 (59.9)	.009
Yes	108 (49.8)	385 (40.1)	
Received disability or workers’ compensation benefits owing to low back pain			
No	148 (68.2)	755 (78.6)	.001
Yes	69 (31.8)	205 (21.4)	
Involved in a legal action owing to low back pain			
No	185 (85.3)	883 (92.0)	.002
Yes	32 (14.7)	77 (8.0)	
Pain catastrophizing score, mean (SD)^a^	24.3 (14.7)	18.4 (12.7)	<.001
Pain self-efficacy score, mean (SD)^a^	32.4 (14.8)	33.4 (14.9)	.41
History of medical conditions			
Herniated disc			
No	149 (68.7)	570 (59.4)	.01
Yes	68 (31.3)	390 (40.6)	
Sciatica			
No	153 (70.5)	440 (45.8)	<.001
Yes	64 (29.5)	520 (54.2)	
Osteoarthritis			
No	151 (69.6)	483 (50.3)	<.001
Yes	66 (30.4)	477 (49.7)	
Osteoporosis			
No	193 (88.9)	819 (85.3)	.16
Yes	24 (11.1)	141 (14.7)	
Hypertension			
No	106 (48.8)	558 (58.1)	.01
Yes	111 (51.2)	402 (41.9)	
Heart disease			
No	200 (92.2)	849 (88.4)	.11
Yes	17 (7.8)	111 (11.6)	
Diabetes			
No	161 (74.2)	792 (82.5)	.005
Yes	56 (25.8)	168 (17.5)	
Asthma			
No	167 (77.0)	698 (72.7)	.20
Yes	50 (23.0)	262 (27.3)	
Depression			
No	97 (44.7)	403 (42.0)	.46
Yes	120 (55.3)	557 (58.0)	
Health-related quality of life SPADE score, mean (SD)^a^	57.9 (7.3)	57.9 (6.7)	>.99
Current use of opioids for low back pain			
No	132 (60.8)	647 (67.4)	.06
Yes	85 (39.2)	313 (32.6)	

Abbreviations: BMI, body mass index (calculated as weight in kilograms divided by height in meters squared); SPADE, sleep disturbance, pain interference with activities, anxiety, depression, and low energy or fatigue.

^a^ Higher scores represent worse clinical status on each of these continuous measures except pain self-efficacy score.

**Figure 2.**
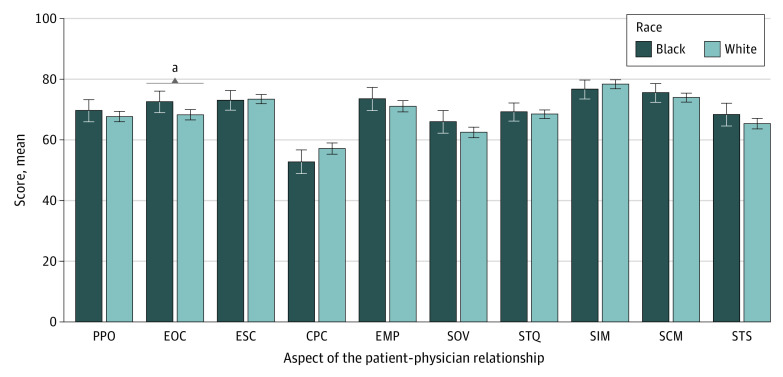
Aspects of the Patient-Physician Relationship According to Race CPC indicates communication about personal circumstances; EMP, physician empathy; EOC, effective and open communication; ESC, emotionally supportive communication; PPO, patient participation and patient orientation; SCM, patient satisfaction with physician communication; SIM, patient satisfaction with physician interpersonal manner; SOV, overall patient satisfaction; STQ, patient satisfaction with technical quality of the physician; STS, patient satisfaction with time spent with the physician; and error bars, 95% CIs. ^a^Statistically significant difference (*P* = .03).

Overall, participants rated the interpersonal manner of the physician as the most favorable aspect of their patient-physician relationship (mean score, 77.6 [95% CI, 76.2-78.9]), and communication about personal circumstances as the least favorable aspect (mean score, 56.0 [95% CI, 54.3-57.7]) (Figure 2). Black and White participants reported similar scores for 9 of 10 aspects of the patient-physician relationship. The only racial difference involved effective and open physician communication, which favored Black participants (mean score, 72.1 [95% CI, 68.8-75.4] vs 67.9 [95% CI, 66.2-69.6]; *P* = .03). However, this was not considered a clinically important difference (d = 0.16 [95% CI, 0.01-0.30]).

**Figure 3.**
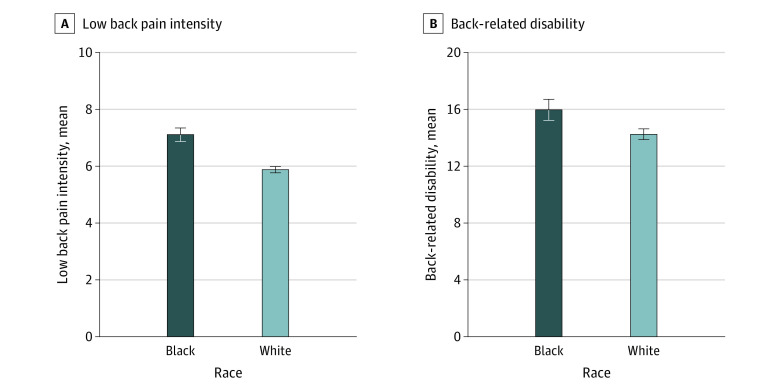
Chronic Pain Outcomes According to Race Error bars indicate 95% CIs.

Pain intensity (mean [SD] score, 6.1 [1.8]) and back-related disability (mean [SD] score, 14.5 [5.7]) were correlated (*r* = 0.46; *P* < .001). Black participants, compared with White participants, reported worse outcomes for pain intensity (mean score, 7.1 [95% CI, 6.8-7.3] vs 5.8 [95% CI, 5.7-6.0]; *P* < .001) and back-related disability (mean score, 15.8 [95% CI, 15.1-16.6] vs 14.1 [95% CI, 13.8-14.5]; *P* < .001) (Figure 3). These racial differences both met the criterion for clinical importance. Racial differences in pain intensity were in the medium to large range (*d* = 0.70 [95% CI, 0.55-0.85]), whereas differences in back-related disability were less pronounced (*d* = 0.30 [95% CI, 0.15-0.45]). Black participants were also at greater risk for severe pain (OR, 3.13 [95% CI, 2.30-4.25]; *P* < .001) and severe back-related disability (OR, 1.61 [95% CI, 1.20-2.17]; *P* = .001). The disease risk score for severe pain among Black participants (mean [SD], 0.50 [0.23]) was greater than among White participants (mean [SD], 0.39 [0.19]; *P* < .001).

**Figure 4.**
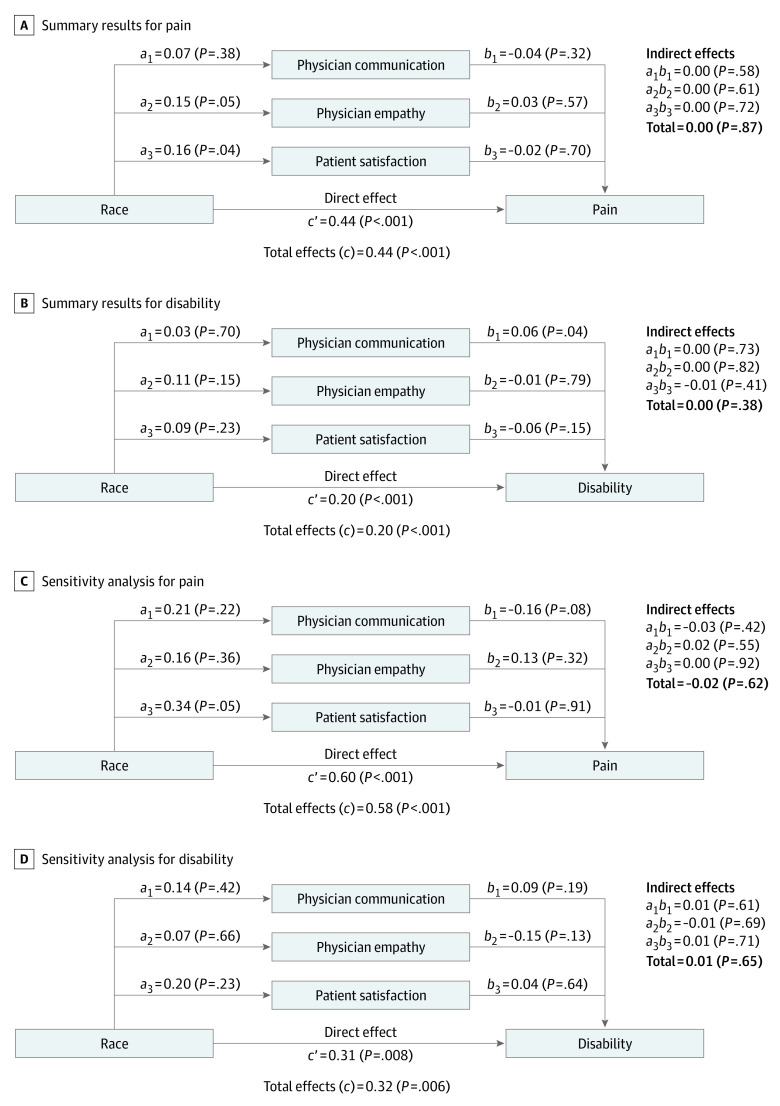
Summary of Mediation Analysis Results a, b, and ab indicate standardized coefficients in the regression model analyses. c’ indicates the direct effect of race with the outcome measures and c indicates the total effects. All values are for Black participants compared with White participants and adjusted for disease risk score. Positive values indicate greater pain or disability and negative values indicate less pain or disability. Total values for indirect effects may not equal the sum of their component values because of rounding.

Despite significant associations between race and each outcome measure, none of the 3 aspects of the patient-physician relationship, either individually or in combination, significantly mediated the observed associations (Figure 4). The percentage of the total association of race with each outcome attributed to the mediator variables was essentially zero. There was a stronger total association of Black race with pain intensity (*c* = 0.44) than with back-related disability (*c* = 0.20). The sensitivity analyses that excluded the disease risk score for potential confounders yielded stronger associations of race with pain intensity (*c* = 0.67) and back-related disability (c = 0.30). These unadjusted results are not presented in this study because the mediation analysis results remained essentially unchanged. The sensitivity analyses involving only participants with a long-term patient-physician relationship involved 310 participants, including 39 Black participants (12.6%) and 271 White participants (87.4%). Despite a smaller sample size, these analyses again demonstrated significant associations of race with pain intensity and back-related disability. Although the associations of Black race with pain intensity (*c* = 0.58) and back-related disability (*c* = 0.32) were magnified in these analyses, the percentage of the total association of race with each outcome attributed to the mediator variables remained essentially zero.

## Discussion

This cross-sectional study found that Black participants experienced clinically important pain disparities compared with White participants. However, Black and White participants differed on many characteristics that may have confounded the associations between race and pain outcomes. Mediation analyses, which adjusted for these potential confounders, demonstrated that the associations of race with disparities in pain and function outcomes were not mediated by physician communication, physician empathy, or patient satisfaction. These findings are somewhat surprising, given that historical evidence points to the potential role of physician bias, stereotyping, and prejudice in the genesis of racial health disparities.^23,24,25^

The socioecological model helps reconcile our findings in light of issues that contribute to racial pain disparities. The model positions patient-physician communication and related factors, such as physician empathy, at the intersection of patient-, physician-, and system-level factors that influence racial pain disparities.^25^ These are often nested within one another, thereby demonstrating the complex nature of the relationships between variables at each level. Our mediation analysis begins to untangle these interrelationships. On a level pertaining to patient factors, we adjusted for 20 potentially important confounders. On another level pertaining to physician factors, we used comprehensive participant-reported data on the patient-physician relationship that are not generally available in clinical settings. Moreover, these data likely reflected candid and unbiased participant assessment of the patient-physician relationship because the physician’s identity was not reported to the registry. The remaining level of the model, not fully addressed in our study, involves the health care system. Optimally, this is exemplified by access to high-quality medical care, including such considerations as availability of health insurance, the health care setting, and access to specialized pain treatment.^25^ Our study considered the health care system only to the degree that all participants had a physician who regularly treated their low back pain. This may explain why Black participants were not undertreated for pain. For example, there was no significant difference in use of opioids for low back pain among Black participants compared with White participants. Nevertheless, it is possible that mediators of racial pain disparities may lie within other aspects of the health care system.

### Limitations

This study has some limitations. Our registry, which uses a digital research platform to collect standardized data from participants in real-world settings across the nation, may offer clinical insights pertaining to the target population.^26^ However, there are potential limitations associated with use of this registry. First, participants were not randomly selected from the US population, and there was a disproportionately high representation of women. Second, the eligibility criteria precluded assessment of the impact of the patient-physician relationship on racial pain disparities in settings used by disadvantaged patients without regular access to medical care. Third, because data on the racial concordance of patients and physicians were not collected, its association with the patient-physician relationship and clinical outcomes could not be assessed. Additionally, cross-sectional studies are generally limited in assessing cause and effect. However, this was not germane to our study because mediation effects were not observed.

## Conclusions

The findings of this cross-sectional study suggest that factors other than the patient-physician relationship were associated with the pain disparities experienced by Black participants. Additional research on systemic factors, such as access to high-quality medical care, including consideration of the availability of health insurance, the health care setting, and access to specialized pain treatment, may identify more promising approaches to mitigate racial pain disparities.
