# Error in Author Degree

**DOI:** 10.1001/jamanetworkopen.2023.37031

**Published:** 2023-09-25

In the Original Investigation titled “Analysis of the Patient-Physician Relationship, Race, and Pain Control and Physical Function Among Adults With Chronic Low Back Pain,”^1^ published June 9, 2022, author Leah Goehring’s degree should have been provided as BS. This article has been corrected.^1^
